# Hematopoietic Sphingosine 1-Phosphate Lyase Deficiency Decreases Atherosclerotic Lesion Development in LDL-Receptor Deficient Mice

**DOI:** 10.1371/journal.pone.0063360

**Published:** 2013-05-20

**Authors:** Martine Bot, Paul P. Van Veldhoven, Saskia C. A. de Jager, Jason Johnson, Niels Nijstad, Peter J. Van Santbrink, Marijke M. Westra, Gerd Van Der Hoeven, Marion J. Gijbels, Carsten Müller-Tidow, Georg Varga, Uwe J. F. Tietge, Johan Kuiper, Theo J. C. Van Berkel, Jerzy-Roch Nofer, Ilze Bot, Erik A. L. Biessen

**Affiliations:** 1 Division of Biopharmaceutics, Leiden Academic Centre for Drug Research, Leiden University, Leiden, The Netherlands; 2 LIPIT, Department of Molecular Cell Biology, K.U. Leuven, Leuven, Belgium; 3 Bristol Heart Institute, Bristol Royal Infirmary, Bristol, England; 4 Department of Pediatrics, Center for Liver, Digestive, and Metabolic Diseases, University Medical Center Groningen, University of Groningen, Groningen, The Netherlands; 5 Experimental Vascular Pathology Group, Department of Pathology, Maastricht University Medical Center, Maastricht, The Netherlands; 6 Department of Medicine, Hematology and Oncology, University Hospital Münster, Münster, Germany; 7 Institute of Experimental Dermatology, University of Münster, Münster, Germany; 8 Center for Laboratory Medicine, University Hospital Münster, Münster, Germany; 9 Department of Internal Medicine, Endocrinology, and Geriatrics, University of Modena and Reggio Emilia, Modena, Italy; French National Centre for Scientific Research, France

## Abstract

**Aims:**

Altered sphingosine 1-phosphate (S1P) homeostasis and signaling is implicated in various inflammatory diseases including atherosclerosis. As S1P levels are tightly controlled by S1P lyase, we investigated the impact of hematopoietic S1P lyase (*Sgpl1^−/−^)* deficiency on leukocyte subsets relevant to atherosclerosis.

**Methods and Results:**

LDL receptor deficient mice that were transplanted with *Sgpl1^−/−^* bone marrow showed disrupted S1P gradients translating into lymphopenia and abrogated lymphocyte mitogenic and cytokine response as compared to controls. Remarkably however, *Sgpl1^−/−^* chimeras displayed mild monocytosis, due to impeded stromal retention and myelopoiesis, and plasma cytokine and macrophage expression patterns, that were largely compatible with classical macrophage activation. Collectively these two phenotypic features of *Sgpl1* deficiency culminated in diminished atherogenic response.

**Conclusions:**

Here we not only firmly establish the critical role of hematopoietic S1P lyase in controlling S1P levels and T cell trafficking in blood and lymphoid tissue, but also identify leukocyte *Sgpl1* as critical factor in monocyte macrophage differentiation and function. Its, partly counterbalancing, pro- and anti-inflammatory activity spectrum imply that intervention in S1P lyase function in inflammatory disorders such as atherosclerosis should be considered with caution.

## Introduction

The lysosphingolipid sphingosine 1-phosphate (S1P) is an important lipid mediator generated from sphingosine upon cell activation and present in plasma and extracellular fluid at high nanomolar concentration [1], [2]. Almost all cells of hematopoietic origin, including platelets, mast cells, neutrophils, erythrocytes and mononuclear cells are able to store and release S1P, presumably via ATP binding cassette transporter C1 (ABCC1) [3]–[5].

A large body of evidence supports a major regulatory role of S1P in lymphocyte proliferation, migration and cytokine secretion [6], [7]. Moreover, S1P and its receptors are critically involved in maintaining proper lymphocyte egress from lymphoid organs [8]–[10]. In fact, S1P gradients between lymphoid organs with low S1P concentration and the circulation, which contains high S1P levels, are a driving force for lymphocyte fluxes [7], [11]. However, the actual regulation of S1P gradients remains elusive, as most cell types are able to generate S1P through ubiquitously expressed sphingosine kinases 1 and 2 [12], [13], and degrade it through S1P lyase or S1P phosphatases 1 and 2 [1]. In addition to its contribution to lymphocyte trafficking, S1P also plays a major role in endothelial integrity and confers protection against tumor necrosis factor (TNF)-α-induced monocyte-endothelial interactions [14], [15]. Furthermore, S1P or analogues thereof are known to inhibit apoptosis in monocytes and bone marrow-derived macrophages [16] and to polarize macrophages towards less inflammatory alternatively activated phenotype [17].

The S1P-induced impairment of T cell trafficking and T cell and macrophage activation may at least in part account for the beneficial effects exerted by this lysosphingolipid in animal models of inflammatory diseases [17]–[20]. Atherosclerosis, the underlying cause of acute cardiovascular syndromes such as myocardial infarction and stroke, is considered as a lipid-driven inflammatory disease. As lymphocyte and macrophage activation within the arterial wall are essential processes in atherosclerotic plaque initiation and progression [21], an involvement of S1P in this inflammatory disorder has been postulated as well. Indeed recent studies by us and others have shown that FTY720, a synthetic S1P analogue, reduced atherogenesis by causing lymphocyte sequestration in lymph nodes, by impairing monocyte penetration into the arterial wall, and by exerting anti-inflammatory effects on macrophages and endothelium [17], [22]. Recently, the sphingosine kinase inhibitor ABC294640 was demonstrated to reduce plasma S1P levels, but did not affect atherosclerosis due to counterbalancing pro- (enhanced activation of dendritic cells and T-cells) and anti-atherogenic effects (endothelial cells activation) [23]. Also, a number of S1P receptors have been implicated in atherosclerosis, such as S1P_2_ and S1P_3_ [24], [25]. Both receptors were demonstrated to be involved in the monocyte/macrophage recruitment and retention, thereby enhancing atherosclerotic lesion development. Taken together, these studies suggest a dual role for S1P and S1P signaling in the development of atherosclerosis, which may result from differences in experimental setup and animal models used. As S1P degradation is an important process in S1P regulation and homeostasis and concomitant fluxes of inflammatory cells, disruption of this process may give more insight on the role of S1P and S1P signaling in atherosclerosis. As mentioned above, S1P lyase (*Sgpl1*) is a key enzyme in intracellular S1P degradation and is mainly involved in cleavage of S1P into fatty aldehydes and phosphoethanolamine. *Sgpl1* is tightly involved in S1P gradient maintenance in lymphoid organs and is expressed on cells of hematopoietic origin [26], [27]. Therefore, we here sought to investigate the impact of impaired S1P degradation and signaling on atherosclerosis in LDLr^−/−^ mice with hematopoietic deficiency of S1P lyase (*Sgpl1^−/−^*).

## Materials and Methods

### Animals

All animal work was approved by the Ethics Committee for Animal Experiments of Leiden University (Permit Number: 09135) and performed in compliance with the Dutch government guidelines. All experiments were conducted in compliance with the Directive 2010/63/EU of the European Parliament. LDL receptor deficient mice (LDLr^−/−^, Jackson Laboratories) on a C57Bl/6 background were bred in the local animal breeding facility. S1P lyase deficient (*Sgpl1^−/−^*) and wild type littermates were obtained by crossing *Sgpl1* ^+/−^ mice, inbred in a C57Bl/6 background, in the animal housing facilities of the University of Leuven [26], [27]. *Sgpl1* ^+/−^ non-inbred mice were generated from gene trapped ES cells (OST 58278) by Lexicon Inc. (Texas). Total body *Sgpl1^−/−^* mice display early lethality [26], [27], and therefore these mice do not survive long enough to develop atherosclerosis. As hematopoietic cells express *Sgpl1*, we applied a model of bone marrow transplantation as described below to investigate the impact of hematopoietic *Sgpl1* deficiency on atherosclerosis in LDLr^−/−^ mice.

### Irradiation and Bone Marrow Transplantation

**Figure 1:**
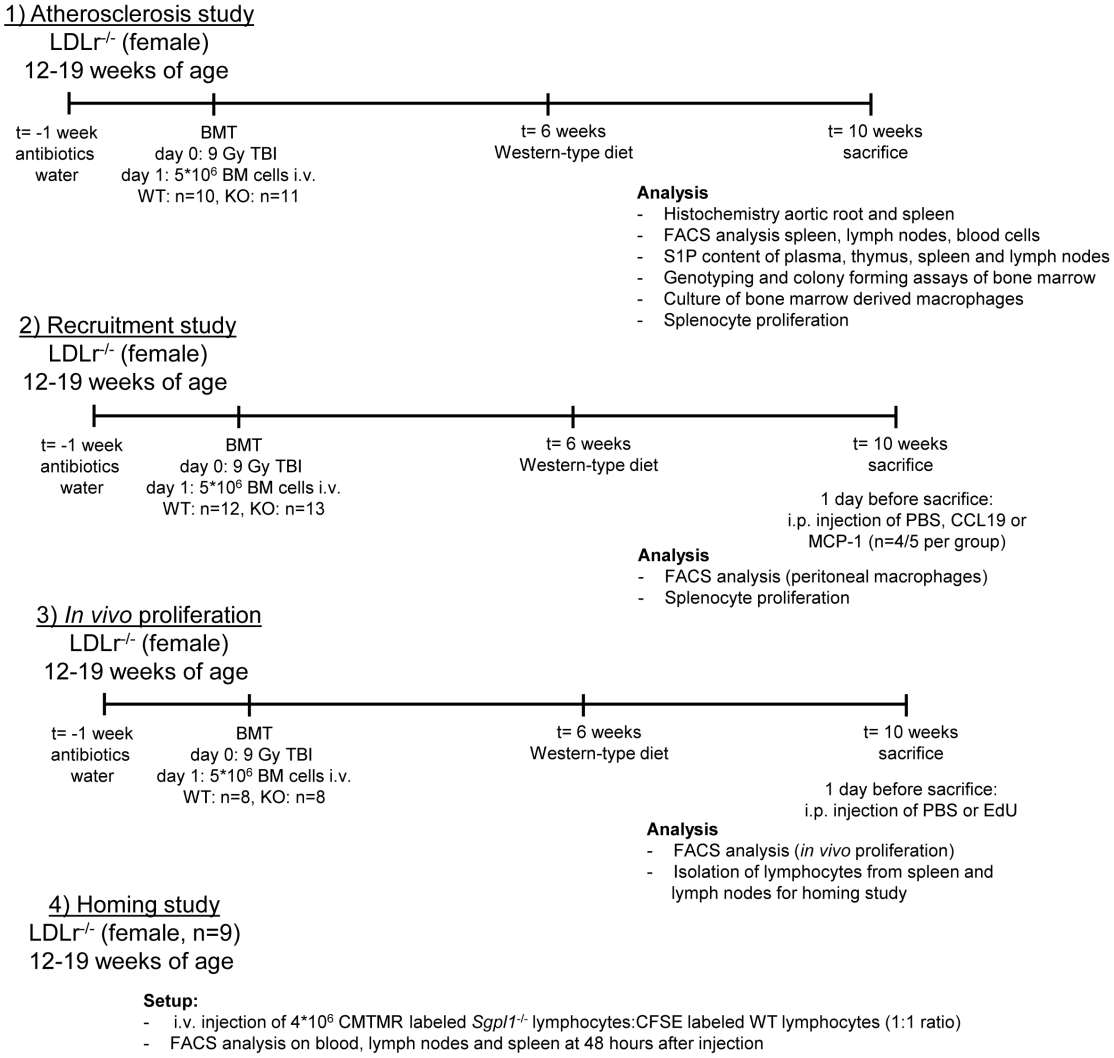
Detailed outline of the experimental setup.

The female LDLr^−/−^ recipients were 12–19 weeks of age (n = 21 for atherosclerotic lesion analysis, n = 25 for recruitment studies and n = 16 for *in vivo* proliferation and macrophage characterization studies, see Figure 1 for a detailed outline of the experiments). One week before bone marrow transplantation and throughout the study female LDLr^−/−^ recipients (12–19 weeks of age) were given ad libitum drinking water supplemented with antibiotics (83 mg/L ciprofloxacin and 67 mg/L Polymixin B sulphate) and 6,5 g/L sugar. To induce bone marrow aplasia, female LDLr^−/−^ mice were exposed to a single dose of 9 Gy (0.19 Gy/min, 200 kV, 4 mA) total body irradiation, using an Andrex Smart 225 Röntgen source (YXLON International, Copenhagen, Denmark) with a 6-mm aluminum filter, one day before transplantation. Bone marrow cell suspensions were isolated from 10–14 day old *Sgpl1^−/−^* and ^+/+^ littermates (anaesthetized using a single subcutaneous injection of ketamine (60 mg/kg, Eurovet Animal Health, Bladel, The Netherlands), fentanyl citrate and fluanisone (1.26 mg/kg and 2 mg/kg respectively, VetaPharma Ltd, Leeds, UK) by flushing the femurs, tibias, humeri, radii and ulnas with phosphate buffered saline (PBS, 150 mM NaCl, 1.5 mM NaH_2_PO_4_, 8.6 mM Na_2_HPO_4_, pH 7.4). Single-cell suspensions were prepared by passing the cells through a 70 µm cells strainer (BD, Breda, The Netherlands) and 5×10^6^ cells were injected into the tail vein of the irradiated recipients.

After bone marrow transplantation mice were housed in sterile filter-top cages and fed a sterile regular chow diet (RM3; Special Diet Services, Witham, United Kingdom) for 6 weeks and a Western type diet containing 0.25% cholesterol and 15% cacaobutter for another 4 weeks (Western diet; Special Diet Services). Throughout the experiment animal weight was monitored.

### Tissue Harvesting

For recruitment studies, mice received intraperitoneal injections of CCL19 (500 ng/mL; Peprotech, Rocky Hill, NJ), MCP-1 (1 µg/mL; Peprotech, Rocky Hill, NJ) or phosphate buffered saline (PBS) 16–24 hours before sacrifice. For *in vivo* proliferation measurement mice received an intraperitoneal injection of EdU (100 µg, Invitrogen, Breda, the Netherlands) or PBS as a control 96 and 24 hours before sacrifice. At sacrifice mice were anaesthetized by subcutaneous injection of ketamine (60 mg/kg, Eurovet Animal Health, Bladel, The Netherlands), fentanyl citrate and fluanisone (1.26 mg/kg and 2 mg/kg respectively, VetaPharma Ltd, Leeds, UK) and bled by orbital exsanguination. Peritoneal leukocytes were isolated by peritoneal lavage with 10 mL of ice-cold PBS, after which the composition of the peritoneal leukocyte populations were analyzed by FACS analysis.

Mice were perfused with PBS via the left cardiac ventricle. Organs were excised and stored on ice for direct use (flow cytometry), fixed in 4% Zinc Formalfixx (Shandon Inc, Thermo Fisher Scientific BV, Breda, The Netherlands) for histological analysis, or snap-frozen in liquid nitrogen for optimal RNA and DNA preservation. Single-cell suspensions were prepared of part of the spleen, mesenteric lymph nodes and thymus. Erythrocytes in blood and spleen suspensions were lysed by hypo-osmotic shock in erythrocyte lysis buffer (0.01 M Tris, 0.83% NH_4_Cl, pH7.2) for 5 minutes on ice.

S1P levels in plasma, thymus, lymph nodes and spleen were quantified as follows: acidic methanolic extracts of plasma or tissues were fortified with the internal standard C_17_-S1P, prepared from C_17_-sphingosine with recombinant human Sphk1, diluted with water and applied to a hydrophobic SPE cartridge. Compounds eluted with methanol, were derivatized with 6-aminoquinolyl-N-hydroxysuccinimidyl carbamate and subjected to normal phase SPE to separate the derivatized sphingoid bases and their phosphate esters. After two selective hydrolysis steps, samples were separated by reversed phase HPLC (Symmetry C18-column 4.6×150; 5 µm; 100 Å; Waters) with an increasing gradient of buffered methanol/acetonitrile coupled to fluorimetric analysis.

### Differential Blood Cell Analysis

Differential blood cell analysis was performed with by automated differential cell count analysis (Sysmex, Goffin Meyvis BV, Etten Leur, The Netherlands) or by flow cytometry (FACSCalibur, FACSCANTO II or LSRII, BD) on whole blood, white blood cells, and single-cell suspensions of spleen, lymph nodes and bone marrow. For each FACS staining 2*10^5^ cells were incubated with antibody dilutions (0.25 µg for each antibody) in PBS plus 1% mouse serum at 4°C. Monoclonal antibodies for flow cytometry were from BD, Breda, The Netherlands (CD4, CXCR4, CD8 and streptavidin-PE), eBioscience, Zoersel-Halle, Belgium (CCR7, CD8, CD19, CD44, CD62L, CD11b, GR1, CD71, CD11c, F4/80, MHCII and CD86), Abcam, Cambridge, UK (CCR2, S1P_1_ [28], goat-anti-rabbit-FITC and donkey-anti-rabbit-PE) or Invitrogen (EdU Alexa Fluor® 488).

### Plasma Cytokine Determination

Mouse plasma cytokines (i.e. IL-6, IL-10, MCP-1, IFN-γ, TNF-α and IL-12p70) were determined by a cytometric bead array (CBA, BD) on a FACSCalibur (BD). Calibration curves were established from standard solutions provided by BD. Analysis of calibration curves and samples was done using BD™ CBA software.

### Splenic T Cell Proliferation and Cytokine Production

T cells were purified from freshly isolated splenocytes by a T lymphocyte enrichment set (BD) or lympholyte-M (CEDARLANE Laboratories Ltd., Burlington, NC, USA). T cell suspensions were washed, resuspended in RPMI1640 (PAA Laboratories, Cölbe, Germany) containing 10% fetal calf serum (FCS, v/v), 2 mM L-glutamine, 100 U/mL penicillin, and 100 µg/mL streptomycin, and 50 µM β-mercaptoethanol (RPMI complete) and seeded at a density of 2×10^5^ cells/well in 96-well plates. Cells were incubated for 40 hours in RPMI or RPMI supplemented with S1P (100 nM; Bio Connect BV, Huissen, The Netherlands), αCD3/αCD28 (2 µg/mL, eBioscience) or concanavalin A (ConA; 2 µg/mL; Sigma, Zwijndrecht, The Netherlands). After 24 hours [^3^H]thymidin (5.0 µCi/well; GE Healthcare, Eindhoven, The Netherlands) was added and after 16 hours cell-associated radioactivity was determined by scintillation spectrometry. IL-2, IL-4, IL-10, IL-12 and INF-γ contents in the supernatant were determined by commercially available ELISA (eBioscience).

### Functional Characterization of Peritoneal and Bone Marrow-derived Macrophages

Peritoneal macrophages (p-mφ) were harvested as described above. Bone marrow-derived macrophages (BM-mφ) were cultured for 7 days in RPMI1640 supplemented with 20% FCS (v/v), 2 mM L-glutamine, 100 U/mL penicillin, and 100 µg/mL streptomycin, 1% non essential amino acids (v/v), 1% pyruvate (v/v), and macrophage colony-stimulating factor (M-CSF). After detachment with 4 mM EDTA, macrophages were resuspended in DMEM (PAA Laboratories) containing 10% FCS (v/v) and 2 mM L-glutamine, 100 U/mL penicillin, and 100 µg/mL streptomycin, and seeded in a 24-well plate at a density of 0.5×10^6^ cells/mL. For experiments macrophages were cultured at a density of 0.5×10^6^ cells/mL and were incubated for 24 hours in the absence or presence of lipopolysaccharide (LPS; 50 ng/mL; *Salmonella Minnesota* R595 (Re); List Biological Laboratories Inc. Campbell, CA) or IL-4 (100 ng/mL [p-mφ] and 10 ng/mL [BM-mφ], Peprotech). IL-6, IL-10, IL-12, MCP-1 and TNF-α contents in medium were determined by commercially available ELISA (eBioscience and BD). Gene expression was quantitatively analyzed on an ABI7500 Fast Real-Time PCR system (Applied Biosystems, Foster City, CA) as described previously [29], with murine hypoxanthine phosphoribosyltransferase (HPRT) as housekeeping gene (Table 1).

**Table 1:** RT-PCR primer sequences for gene expression analysis of peritoneal macrophages and bone marrow-derived macrophages.

Gene	Source	forward primer (5′-3′)	reverse primer (5′-3′)
*Arginase 1*	NM_007482	GGTTCTGGGAGGCCTATCTTACA	TCTTCACCTCCTCTGCTGTCTTC
*CCL3*	NM_011337	GCCACATCGAGGGACTCTTCA	GATGGGGGTTGAGGAACGTG
*CCR2*	NM_009915	AACTGTGTGATTGACAAGCACTTAGAC	TGACAGGATTAATGCAGCAGTGT
*CCR5*	NM_009917	GACTGTCAGCAGGAAGTGAGCAT	CTTGACGCCAGCTGAGCAA
*IL-1α*	NM_010554	GCGCTCAAGGAGAAGACCAG	TGATACTTTTCCAGAAGAAAATGAGG
*IL-1RA*	NM_031167	TTCATAGTGTGTTCTTGGGCATC	CGCTTGTCTTCTTCTTTGTTCTTG
*IL-6*	M20572	GAAGAATTTCTAAAAGTCACTTTGAGATCTAC	CACAGTGAGGAATGTCCACAAAC
*IL-10*	NM_010548	TCCCCTGTGAAAATAAGAGCA	ATGCAGTTGATGAAGATGTCAAA
*IL-12 p35*	NM_008351	AGTGAAAATGAAGCTCTGCATCC	GATAGCCCATCACCCTGTTGA
*IL-12 p40*	NM_008352	GATTCAGACTCCAGGGGACA	GGAGACACCAGCAAAACGAT
*iNOS*	NM_010927	CCTGGTACGGGCATTGCT	GCTCATGCGGCCTCCTTT
*MCP-1*	M19681	GCATCTGCCCTAAGGTCTTCA	TTCACTGTCACACTGGTCACTCCTA
*TNF-α*	X02611	GCCAGCCGATGGGTTGTA	AGGTTGACTTTCTCCTGGTATGAGA
*HPRT*	NM_013556	TTGCTCGAGATGTCATGAAGGA	AGCAGGTCAGCAAAGAACTTATAG

### Functional Characterization of Bone Marrow

Bone marrow cells of the recipient LDLr^−/−^ mice were flushed from tibias and femurs with PBS and filtered through a cell-strainer. Red blood cells were lysed with lysis buffer (0.15 M NH_4_Cl, 1 mM KHCO_3_, 0.1 mM Na_2_-EDTA, pH 7.3). Bone marrow cells were incubated with the appropriate antibodies in PBS containing 5% calf serum for 30 min on ice in a total volume of 50 µL after which cells were washed with PBS containing calf serum. For functional characterization, bone marrow cells were incubated with anti-murine B220 (Caltag, San Francisco, CA) or anti-murine CD34, CD41, Ter119, Gr1, CD14, Sca1, CD117 (BD Pharmingen) and analyzed with flow cytometry (FACSCalibur, BD). Data were analyzed by CellQuest software. For colony assays, primary mouse bone marrow cells (10^4^/ml methylcellulose) (Stem cell technologies, M3434) were seeded in 3 cm dishes and cultivated for 8–11 days. Colonies were counted and categorized according to their morphology.

### In vivo Splenocyte Homing

Part of the spleen and the mesenteric lymph nodes of the recipient LDLr−/− mice were isolated and for homing/migration experiments of lymphocytes, single cell suspensions of spleen lymphocytes from *Sgpl1^−/−^* chimeras (labeled for 30 min with 20 µM orange-fluorescent tetramethyl-rhodamine [CMTMR], Invitrogen) and wild type controls (labeled for 15 min with 2 µM carboxyfluorescein diacetate succinimidyl ester [CFSE], Invitrogen) were intravenously injected in the tail vein of a separate set of LDLr^−/−^ mice at a 1∶1 ratio (4×10^6^ labeled splenocytes in total) [30]. After 48 hours presence of CFSE and CMTMR labeled spleen lymphocytes in spleen and lymph nodes was examined by flow cytometry and histological analysis.

### Histological and Morphometric Analysis

The aortic root of mice receiving either *Sgpl1^−/−^* or wild type control bone marrow was excised for analysis of spontaneous atherosclerosis, embedded in Tissue-Tek and transverse 10 µm cryosections throughout the aortic valve area were prepared. Sections were stained with Oil-Red-O and hematoxylin (Sigma). Cross-sections with maximal stenosis were used for morphometric analysis on a DM-RE microscope with Leica Qwin image analysis software (Leica Microsystems B.V., Rijswijk, the Netherlands). Corresponding sections were stained with antibodies directed against mouse macrophages (monoclonal mouse IgG_2a_, clone MOMA-2, dilution 1∶50; Sigma Diagnostics, St. Louis, MO) and lymphocytes (CD3 clone SP7, dilution 1∶150, Immunologic, Duiven, The Netherlands). For macrophage phenotype staining, endogenous peroxidase activity was first inhibited by incubating cryosections with 0.3% (v/v) hydrogen peroxide in methanol. After blocking with 1% (w/v) bovine serum albumin (BSA) in PBS, sections were incubated with either purified rat monoclonal antibody against mouse macrophages (F4/80) (Abcam) at 20 µg/mL, goat anti-CD163 (Santa Cruz, UK) at 2 µg/mL, rabbit anti-CCR2 (Abcam) at 10 µg/mL, rabbit anti-iNOS (Abcam) at 10 µg/mL, rabbit anti-MMP-14 (Abcam) at 10 µg/mL or rabbit anti-TIMP-3 (Abcam) at 2.5 µg/mL in 1% (w/v) BSA in PBS. Sections were then incubated with AlexaFlour-488 conjugated secondary antibodies (Invitrogen, UK) diluted 1∶200 in 1% (w/v) BSA in PBS. Sections were mounted with Vectashield containing DAPI (Vector Laboratories, UK) to reveal the nuclei. The relative area stained positive for each of the antibodies was quantified by computerized image analysis (Image Pro Plus, Media Cybernetics, Carlsbad, USA), and was expressed as a percentage of the total atherosclerotic plaque area. A negative control, where the primary antibody was replaced with the relevant IgG at the same dilution, was always included. Transverse 5-µm cryosections were prepared from spleen and stained for CD3.

### Genotyping

Genomic DNA was isolated from bone marrow and bone marrow-derived macrophages by chromatography over DNA extraction columns (Qiagen, Venlo, the Netherlands) and used for verification of the efficacy of bone marrow repopulation after transplantation. Repopulation rate was determined by use of a PCR calibration curve using DNA isolated from wild type and *Sgpl1^−/−^* bone marrow in ratios from 100%:0%, 90%:10%, 80%:20% etc. Primer sets used for the wild type S1P lyase alleles (forward 5′- TGATAGGGCTGAAAACCACTG and reverse 5′- TCAGAAGCAAAACTGCCTTG) and the mutated alleles, containing a β-geo insertion, (forward 5′-CGAATACCTGTTCCGTCATAGC and reverse 5′-ACCACTACCATCAATCCGGTAG).

### Statistical Analysis

Values are expressed as mean ± SEM. A 2-tailed Student’s t-test was used to compare individual groups of animals. To determine significance of the relative mRNA expression levels, statistical analysis was performed on ΔCt values.

## Results

### Assessment of Chimerism and S1P Levels in Hematopoietic *Sgpl1^−/−^* Chimeras

**Figure 2:**
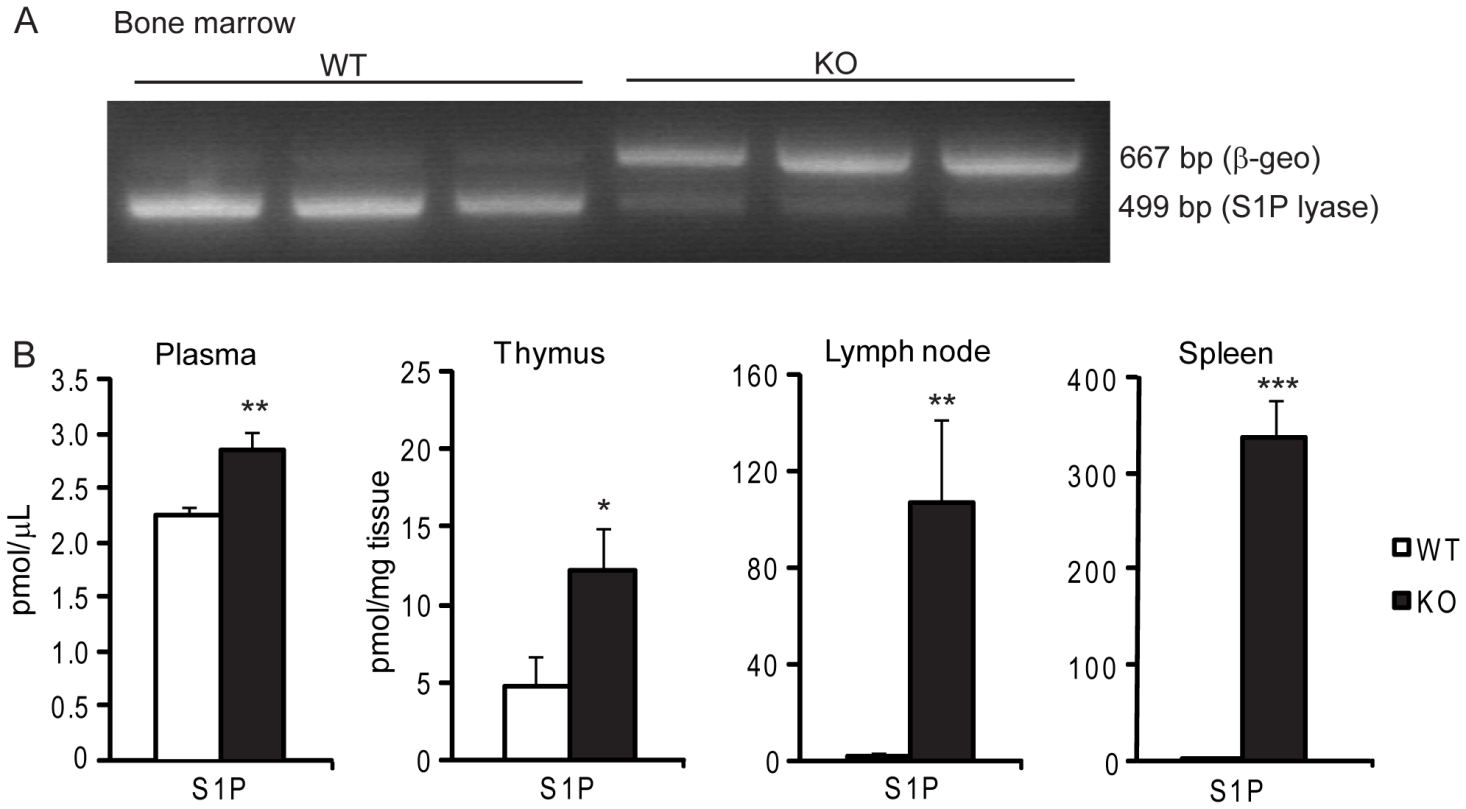
Recipient bone marrow genotyping and S1P concentrations. Genotyping of the bone marrow (A) showed >90% repopulation of the bone marrow for *Sgpl1*. (B) Effect of hematopoietic *Sgpl1^−/−^* on plasma S1P concentration and S1P content of thymus, lymph node, and spleen. White bars: wild-type (WT), black bars: *Sgpl1^−/−^* (KO) chimeras. Data are represented as mean +/− SEM*^−/−^*, data from n = 10–11 per group in total. *P<0.05, **P<0.01, ***P<0.001 (WT versus KO).

To induce hematopoietic deficiency of S1P lyase, lethally irradiated LDLr^−/−^ mice were reconstituted with either *Sgpl1^−/−^* bone marrow or bone marrow isolated from wild type littermates. Bone marrow from *Sgpl1^−/−^* transplanted animals showed >90% repopulation of *Sgpl1^−/−^* bone marrow (Figure 2A). *Sgpl1^−/−^* chimerism did not affect body weight. Hematopoietic *Sgpl1* ^−/−^ led to a profound increase in S1P content in spleen (90-fold, P<0.001) and lymph nodes (47-fold, P<0.01), while total S1P levels in thymus (2.2-fold, P<0.05) and plasma (1.25-fold, P<0.01) were only modestly elevated (Figure 2B).

### Hematopoietic *Sgpl1^−/−^* Chimeras Promotes Lymphopenia

**Figure 3:**
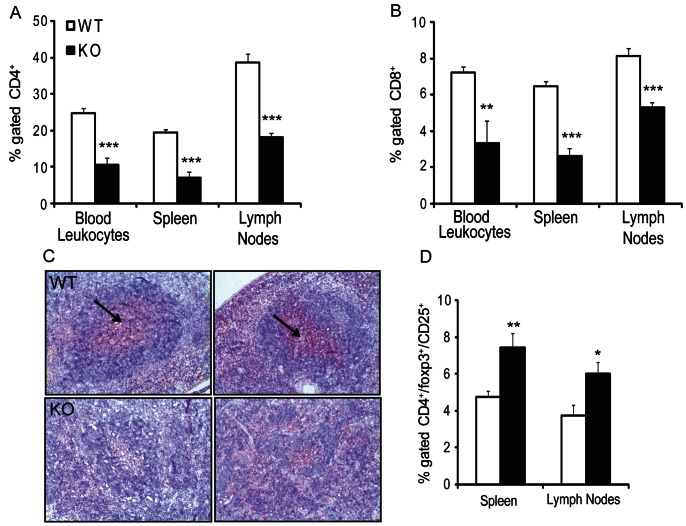
Effect of hematopoietic *Sgpl1^−/−^* on T cell numbers. (A,B) *Sgpl1^−/−^* chimeras show reduced CD4^+^ and CD8^+^ T cell numbers in blood, spleen and lymph nodes. (C) CD3 staining shows absence of normal germinal centre morphology in spleens of *Sgpl1^−/−^* chimeras (scale bar: 100 µm, arrows indicate germinal centers in spleens of WT transplanted mice). (D). *Sgpl1^−/−^* chimeras show a relative increase in regulatory T cells in spleen and lymph nodes. White bars: wild-type (WT), black bars: *Sgpl1^−/−^* (KO) chimeras. Data are represented as mean ± SEM, n = 10–11. *P<0.05, **P<0.01, ***P<0.001 (WT versus KO).

In agreement with previous findings after pharmacological (2-acetyl-4-tetrahydroxybutylimidazole (THI)) [11] or genetic (*Sgpl1*-deficient mice) [27], [31] interruption of *Sgpl1* function, blood lymphocyte counts were decreased from 3.2 to 1.1*10^6^ cells/mL (−63%, P<0.001) in mice with hematopoietic *Sgpl1*-deficiency. *Sgpl1^−/−^* chimeras had sharply reduced CD4^+^ T cell levels in blood, lymph nodes, spleen (>−60%, P<0.001, Figure 3A). CD8^+^ T cells showed a similar pattern with 50–60% reductions in blood, lymph nodes and spleen (Figure 3B, P<0.005). In agreement, immunohistochemistry revealed equally diminished spleen CD3^+^ T cell contents and aberrant germinal center morphology (Figure 3C). Surprisingly, and in contrast to previous observations after systemic S1P lyase inhibition by THI treatment or by using the S1P analogue FTY720, no accumulation of T cells was evident in lymph nodes. Regulatory T cell (CD4^+^/foxp3^+^/CD25^+^) numbers in spleen and lymph node were increased in *Sgpl1^−/−^* chimeras (7.4% versus 4.8% for control chimeras in spleen and 6.0% versus 3.7% for control chimeras in lymph nodes, respectively; both P<0.02) (Figure 3D). Hematopoietic *Sgpl1* deficiency did not noticeably influence total B cell (CD19) numbers in blood and lymph nodes.

### Perturbed T Lymphocyte Proliferation and Trafficking in *Sgpl1^−/−^* Chimeras

**Figure 4:**
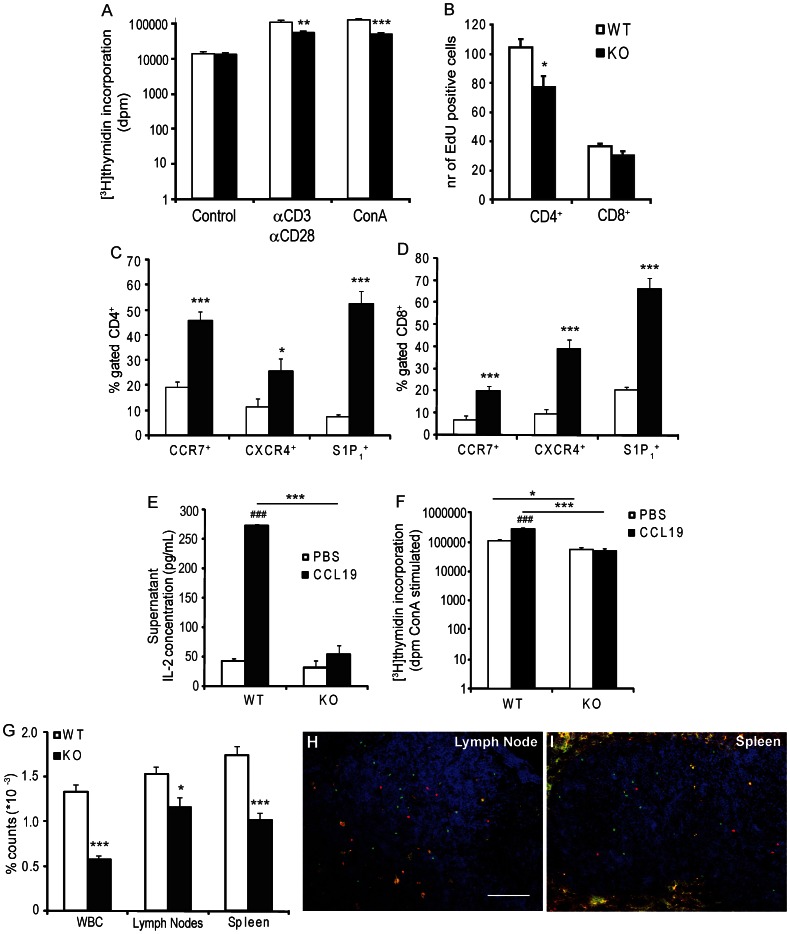
Effect of hematopoietic S1P lyase deficiency on T-cell proliferation and migration. (A) The mitotic response of T cells isolated from *Sgpl1^−/−^* chimeras to αCD3/αCD28 (2 µg/mL) or ConA (2 µg/mL) challenge is markedly decreased compared to *Sgpl1^+/+^* transplanted mice (n = 8 per group). (B) *In vivo* proliferation of CD4^+^ T cells as measured by EdU incorporation is also significantly decreased in the *Sgpl1^−/−^* chimeras (n = 8 per group). (C, D) CCR7, CXCR4 and S1P_1_ positive cells were overrepresented in spleen CD4^+^ and CD8^+^ T-cell subsets of *Sgpl1^−/−^* (n = 10–11 per group). (E) Splenocytes of CCL19-challenged *Sgpl1^+/+^* transplanted mice but not *Sgpl^−/−^* chimeras show ConA induced IL-2 release, which corresponds to reduced proliferation (n = 4 per group) (F). (G) Flow cytometry analysis of lymphoid organs and circulation at 48 hours after injection of a 1∶1 mixture of CMTMR labeled *Sgpl1^−/−^* versus CFSE labeled wild type lymphocytes into LDLr^−/−^ revealed a reduced presence of *Sgpl1^−/−^* lymphocytes in spleen, lymph nodes and blood (WBC, white blood cells) (n = 9). This reduced homing was confirmed by fluorescence microscopic analysis of lymph node (H) and spleen (I), (scale bar: 100 µm, *Sgpl1^−/−^* lymphocytes in red, wild type lymphocytes in green). White bars: wild-type (WT), black bars: *Sgpl1^−/−^* (KO) chimeras. Data are represented as mean ± SEM. *P<0.05, **P<0.01, ***P<0.001 (WT versus KO), ^###^P<0.001 (PBS versus CCL19).

The observed effects on T cells led us to investigate consequences of *Sgpl1* deficiency for the proliferative and mitogenic capacity of lymphocytes. Stimulation of spleen lymphocytes with either αCD3/αCD28 (2 µg/mL) or ConA (2.0 µg/mL) led to a potent mitogenic response in cells from wild type but not *Sgpl1^−/−^* transplanted animals (control: WT = 10154±1344 versus KO = 9671±969 dpm, P = NS; αCD3/αCD28: WT = 74737±9671 versus KO = 38860±3911 dpm, P<0.01 and ConA: WT = 87928±5200 versus KO = 34963±3627 dpm, P<0.001, Figure 4A). In keeping, splenic CD4^+^ T cell proliferation *in vivo* as measured by EdU^+^ cell content was significantly reduced (P<0.05, Figure 4B).

Second, we investigated effects on S1P dependent lymphocyte trafficking, which is thought to be mediated via S1P_1_ receptor. Previously, S1P analogues were shown to quench S1P_1_ expression [9]. Contrary to our expectation, however, S1P_1_^+^ T-cells were overrepresented within the CD4^+^ and CD8^+^ population in blood, lymph nodes and spleen, while S1P_1_ mean fluorescence on a per cell basis did not change. Interestingly, CD4^+^ and CD8^+^ T cell populations in *Sgpl1^−/−^* bone marrow transplanted animals both displayed a striking enrichment in migration markers CCR7 and CXCR4 (Figure 4C, 4D). However, despite the enrichment in CCR7^+^ CD4 and CD8 T cells, the CCR7 migratory response in *Sgpl1^−/−^* chimeras was blunted as illustrated by the complete failure of splenocytes from CCL19 treated mice to secrete IL-2 and IL-4 (Figure 4E and data not shown). Moreover, while T-cells of CCL19-challenged *Sgpl1^+/+^* transplanted mice showed an increased proliferative capacity, this increased response was completely absent in *Sgpl1^−/−^* chimeras (control: WT = 62845±13983 versus KO = 31421±2719 dpm, CCL19: WT = 110329±14903 versus KO = 35464±5634 dpm, Figure 4F).

The most direct proof for intrinsic (thus S1P gradient independent) migratory defects of T cells was provided by a T cell trafficking study in *Sgpl1^−/−^* chimeras. T cell fluxes across secondary lymphoid organs are dependent on S1P gradients, as well as by homeostatic chemokine receptors such as CCR7. Flow cytometric analysis of lymphoid organs and blood at 48 hours after adoptive transfer of an 1∶1 mixture of CMTMR labeled *Sgpl1^−/−^* versus CFSE labeled wild type splenocytes into LDLr^−/−^, indicated a reduced presence of *Sgpl1^−/−^* cells in spleen (−42%), lymph nodes (−25%) and blood (−57%, P<0.05, Figure 4G). The reduced homing capacity of *Sgpl1^−/−^* T cells was confirmed by fluorescence microscopy analysis of lymph node and spleen (Figure 4H, 4I). In summary, these data indicate that T cell proliferation and migration are perturbed in *Sgpl1^−/−^* chimeras, which may impact the development of atherosclerosis.

### Increased Monocyte and Neutrophil Numbers in *Sgpl1^−/−^* Chimeras

**Figure 5:**
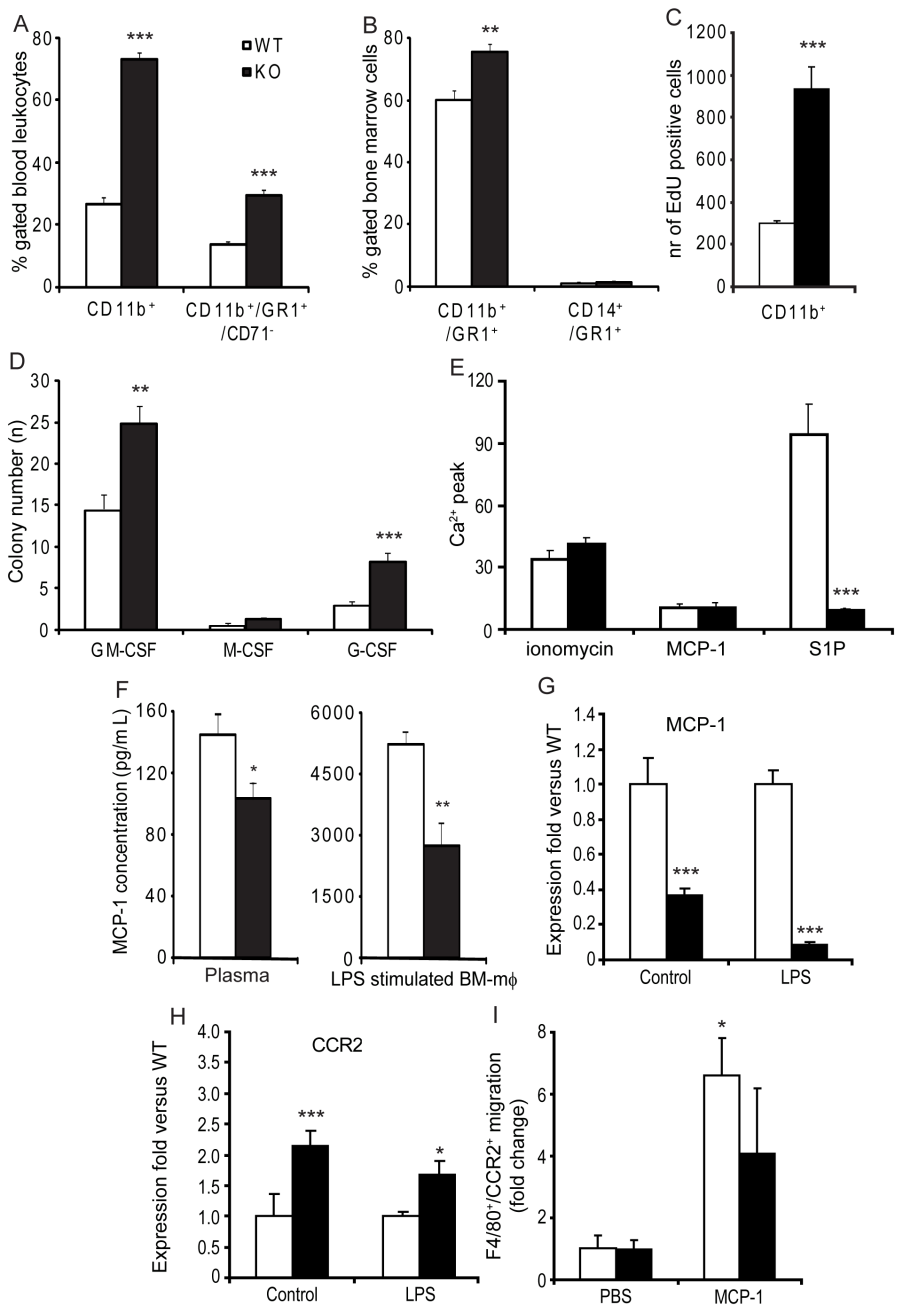
Altered myelopoiesis in *Sgpl1^−/−^*chimeras. (A) *Sgpl1^−/−^*chimeras show increased CD11b^+^ (monocytes) and CD11b^+^Gr1^+^CD71^−^ neutrophil counts in the circulation (n = 10–11 per group). (B) In bone marrow the increase in neutrophils is already noticeable at the level of the CD11b^+^/Gr1^+^ precursors, while this is not the case for the CD14^+^/GR1^+^ monocytes (n = 10–11 per group). (C) *In vivo* proliferation of CD11b^+^ cells as measured by EdU incorporation is 3-fold increased in *Sgpl1^−/−^* chimeras (n = 8 per group). (D) Growth-stimulation of bone marrow cells indicated an increased colonigenic capacity of *Sgpl1^−/−^* cells in response to G-CSF and GM-CSF, while no effect for M-CSF was seen (n = 3 per group). (E) Bone marrow-derived macrophages of *Sgpl1^−/−^*chimeras are unresponsive to S1P stimulus as assessed by Ca^2+^-flux measurement, while the Ca^2+^ response to ionomycin and MCP-1 remained unaltered (n = 3 per group). (F). Analysis of plasma and bone marrow-derived macrophage (BM-mφ) supernatants pointed to reduced MCP-1 generation in *Sgpl1^−/−^*chimeras (plasma: n = 10–11 per group, BM-mφ: n = 5 per group). Reduced MCP1 expression by BM derived macrophages of *Sgpl1^−/−^* chimeras was corroborated at an mRNA levels (G), while conversely expression of CCR2 was increased (H) in bone marrow-derived macrophages (n = 10 per group). However, in a model of mild peritonitis, MCP-1 triggered migration of CCR2^+^ inflammatory macrophages (F4/80, P<0.05) in *Sgpl1^+/+^* transplanted mice but not *Sgpl1^−/−^* chimeras (n = 4–5 per group) (I). White bars: wild-type (WT), black bars: *Sgpl1^−/−^* (KO) chimeras. Data are represented as mean ± SEM. *P<0.05, **P<0.01, ***P<0.001 (WT versus KO).

In the second part of this study we focused on effects of hematopoietic S1P lyase deficiency on the myeloid lineage. Much to our surprise both total blood monocyte and neutrophil counts were markedly elevated in *Sgpl1* ^−/−^ chimeras as assessed by differential cell count analysis (0.2 to 0.6*10^6^ cells/mL and 0.5 to 1.8*10^6^ cells/mL respectively, P<0.005) and by flow cytometry (CD11b^+^ monocytes: +2.7-fold, P<0.001; CD11b^+^/GR1^+^/CD71^−^ neutrophils: +2.2-fold, P<0.001) (Figure 5A). CD11b^+^/GR1^+^ granulocyte precursors in bone marrow were elevated (+27%, P<0.001), while no effects were seen in CD14^+^/GR1^+^ macrophage precursors (Figure 5B). The combined monocytosis/neutrophilia can at least in part be attributed to increased *in vivo* monocyte proliferation (P<0.001, Figure 5C) and augmented granulocyte macrophage-colony stimulating factor (GM-CSF) and granulocyte-colony stimulating factor (G-CSF) dependent myelopoiesis of bone marrow cells in *Sgpl1^−/−^* chimeras (P<0.005, Figure 5D).

To evaluate the responsiveness of these macrophages to different stimuli, we have assessed intracellular Ca^+^-fluxes as a direct measure of S1P and chemokine receptor activation, as chemokines receptors and its ligands such as MCP-1 are important pathways for monocyte and macrophage recruitment in atherosclerosis. Macrophages from *Sgpl1* ^−/−^ chimeras were unable to respond to S1P, whereas activation by MCP-1 and the Ca ionophore ionomycin remained unaltered (Figure 5E). However, plasma MCP-1 levels, which drive stromal monocyte egress [32], were decreased in *Sgpl1^−/−^* chimeras, as was MCP-1 production at protein and mRNA level in basal as well as LPS stimulated bone-marrow-derived macrophages from *Sgpl1^−/−^* chimeras (Figure 5F, 5G). Conversely, bone marrow-derived macrophages had increased CCR2 expression in *Sgpl1^−/−^* macrophages at baseline and after LPS stimulation (Figure 5H), which may either result from hampered differentiation or from altered polarization. A study of peritoneal leukocyte influx in a model of MCP-1 induced peritonitis, in which macrophages are recruited to the peritoneal cavity upon MCP-1 injection which mimics macrophage recruitment to the atherosclerotic plaque, revealed as expected an increased presence of F4/80^+^/CCR2^+^ macrophages (P<0.05) in the peritoneal cavity of the control mice. MCP-1 elicited influx was at least partially blunted in *Sgpl1^−/−^* chimeras (Figure 5I) despite upregulated monocyte CCR2 expression and monocytosis in *Sgpl1^−/−^* chimeras. Thus while *Spgl1* deficient chimeras showed increased circulating monocyte numbers, this subset was desensitized to MCP1 dependent chemotaxis.

### *Sgpl1−/−* Deficient Macrophages are Polarized Towards a Classically Activated Phenotype

**Figure 6:**
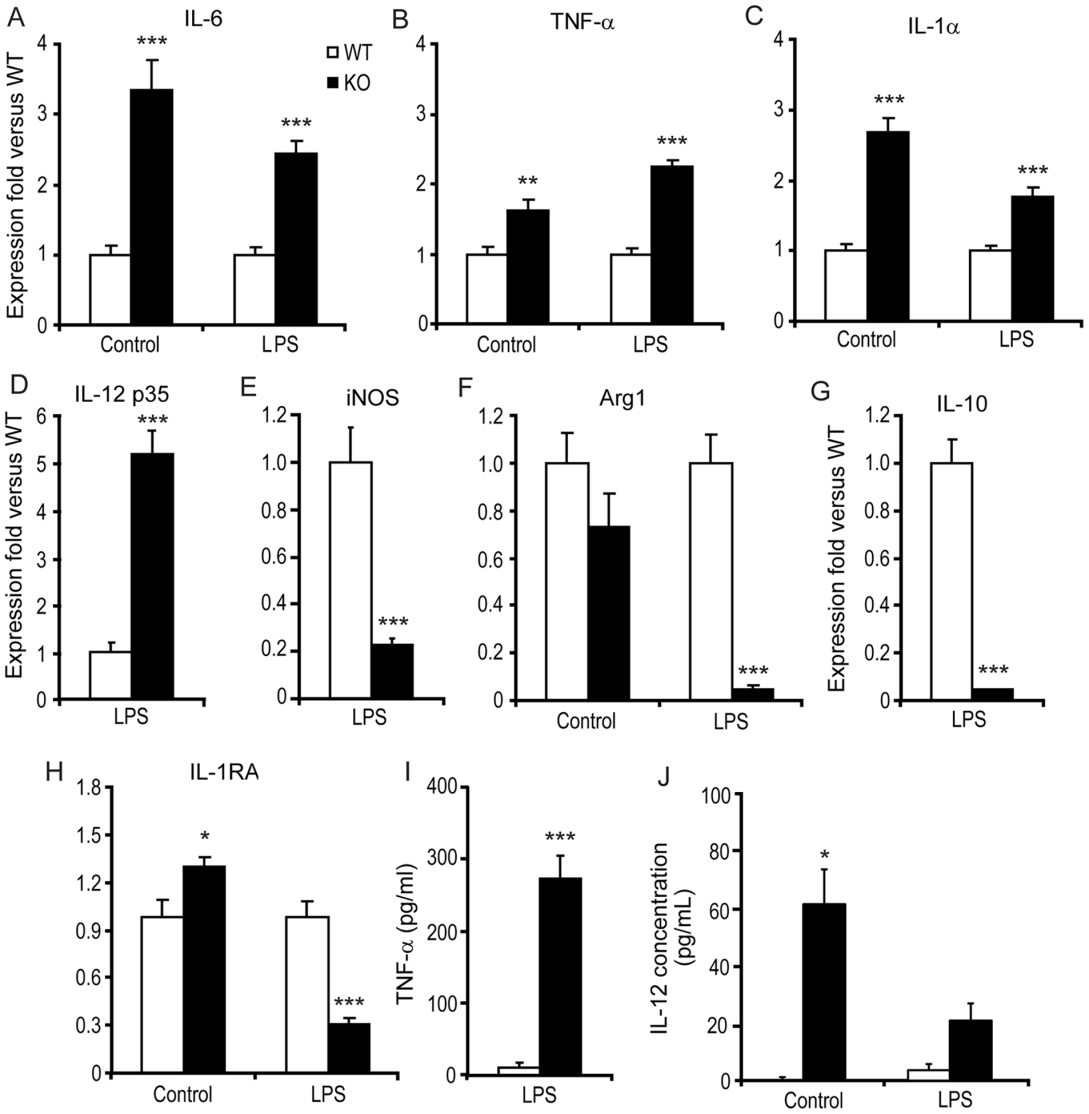
Characterization of macrophage phenotype in *Sgpl1^+/+^* transplanted mice versus *Sgpl1^−/−^* chimeras. Gene expression analysis of bone marrow derived macrophages indicated a generally pro-inflammatory phenotype of *Sgpl1^−/−^* macrophages as judged by the increased expression of the markers IL-6 (A), TNF-α (B), IL-1α (C) and IL-12 (D), while in contrast iNOS expression, another marker of a pro-inflammatory “classically activated” macrophage was sharply decreased (E). Two markers of the anti-inflammatory macrophage phenotype, notably arginase 1 (Arg1) (F) and IL-10 (G) were almost blunted in LPS primed macrophages. Expression of the established M2 marker IL-1RA (H) was increased in control macrophages, while LPS stimulation caused a shift towards downregulated expression in *Sgpl1^−/−^* macrophages. (I) In addition, LPS-induced TNF-α secretion was increased in BM-mφ of *Sgpl1^−/−^* chimera mice. (J) Furthermore, LPS slightly induced the release of the pro-inflammatory IL-12 in control macrophages. The endogenous levels of IL-12 were already higher in *Sgpl1^−/−^* macrophages; however in contrast to control macrophages, LPS did not enhance IL-12 production of these macrophages. White bars: wild-type (WT), black bars: *Sgpl1^−/−^* (KO) chimeras. Data are represented as mean ± SEM, gene expression: n = 10 per group, cytokine analysis: n = 5 per group. *P<0.05, **P<0.01, ***P<0.001 (WT versus KO).

S1P_1_ agonists were previously shown by us and others to favor an anti-inflammatory macrophage phenotype [17], [33]. On the other hand, increased GM-CSF response, as observed in our study, has been shown to favour polarisation of macrophages to a pro-inflammatory phenotype [34], [35]. Therefore, we investigated whether *Sgpl1* deficiency has impacted macrophage activation. Expression analysis on *Sgpl1* ^−/−^ versus *Sgpl1^+/+^* BM-derived macrophages showed a pro-inflammatory phenotype as illustrated by increased expression of pro-inflammatory cytokines IL-6, TNF-α and IL-1α (P<0.001, Figure 6A–D). Paradoxically, expression of another pro-inflammatory macrophage marker, inducible nitric oxide synthase (iNOS) was significantly reduced (P<0.001, Figure 6E). The increase in M1-phenotype marker expression was paralleled by a downregulation of “alternative activation” markers, such as arginase 1 and IL-10 (Figure 6F, 6G), while IL-1 receptor antagonist (IL-1RA) showed a dual response with reduced expression upon LPS stimulation and increased expression in control macrophages (Figure 6H). Further support for a pro-inflammatory macrophage phenotype of *Sgpl1^−/−^* macrophages was provided by increased LPS-induced TNF-α secretion of *Sgpl1^−/−^* BM-derived macrophages and augmented IL-12 secretion by *Sgpl1^−/−^* peritoneal macrophages (Figure 6I, 6J). Concluding, hematopoietic *Sgpl1^−/−^*deficiency results in a more pro-inflammatory macrophage phenotype as compared to control macrophages.

### Hematopoietic *Sgpl1^−/−^* Chimeras Show Reduced Atherosclerosis

**Figure 7:**
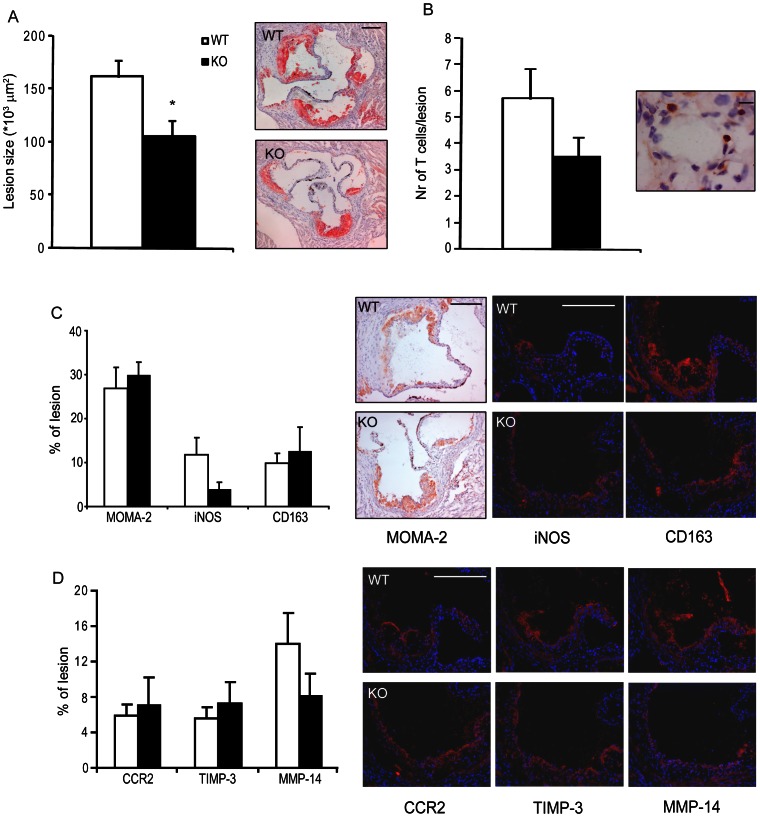
Effect of *Sgpl1^−/−^* chimerism on aortic root lesion size and morphology. (A) Lesion analysis of aortic root lesions shows that absence of hematopoietic S1P lyase decreases atherosclerotic lesion progression compared to control animals by 35% (scale bar: 250 µm). (B) A trend was observed towards a decreased T-cell content in the aortic root (P = 0.09) (scale bar: 10 µm). (C) Despite the observed systemic monocytosis, immunohistochemical analysis did not demonstrate any changes in macrophage content as demonstrated by a MOMA-2 staining and the general macrophage marker CD163. A trend was observed towards decreased plaque expression of the M1 macrophage marker iNOS in *Sgpl1^−/−^* chimeras (P = 0.055) (scale bar: 250 µm, nuclear DAPI staining in blue, macrophage marker staining in red). (D) Macrophage polarization markers such as CCR2 (freshly invaded monocytes) and TIMP3 and MMP14 (foam cells) were unchanged (scale bar: 250 µm, nuclear DAPI staining in blue, macrophage marker staining in red). White bars: wild-type (WT), black bars: *Sgpl1^−/−^* (KO) chimeras, n = 10–11 per group. Data are represented as mean ± SEM. *P<0.05 (WT versus KO).

To determine the effects of hematopoietic *Sgpl1^−/−^*deficiency on atherosclerotic lesion development, Oil Red O-stained aortic root lesions of Western diet fed LDLr^−/−^ chimeras were analyzed. Morphometric quantification revealed that plaque size of the *Sgpl1^−/−^* transplanted mice was significantly decreased as compared to wild type transplanted mice (1.05*10^5^ µm^2^ versus 1.62*10^5^ µm^2^; P = 0.02) (Figure 7A). In concordance with the reduced T-cell migration, hematopoietic *Sgpl1^−/−^* chimeras had borderline significantly reduced CD3^+^ T cell content in lesions (P<0.09) (Figure 7B), however this may be representative for plaque stage and it remains to be established whether this is a causal effect. MOMA positive macrophage presence in plaques from *Sgpl1^−/−^* chimeras did not differ significantly from plaques of *Sgpl1^+/+^* transplanted controls, a surprising finding in view of the monocytosis in the former mice (Figure 7C). In line with the aforementioned reduction in iNOS expression by *Sgpl1^−/−^* macrophages *in vitro*, a strong trend was observed towards decreased iNOS content in lesions of *Sgpl1^−/−^* chimeras (Figure 7C). Analysis of other macrophage markers such as CD163, CCR2 (freshly invaded macrophages), TIMP-3 and MMP-14 (foam cells) [32] did not reveal major differences in plaque expression (Figure 7D). Also, the ratio between TIMP-3 and MMP-14, which is low in highly proteolytic macrophages that can affect plaque stability [36], was also not significantly affected (WT: 0.8±0.3 versus *Sgpl1^−/−^*: 2.2±0.8, P = NS).

## Discussion

S1P signaling is instrumental in the pathogenesis of several inflammatory diseases and recent studies established the atheroprotective activity of a synthetic sphingosine mimetic, FTY720, in a number of mouse models [17], [22]. S1P receptors have also been investigated in disease models of atherosclerosis suggesting a generally protective effect of receptor deficiency [24], [25], [37]. Based on these data, the effects of S1P on cardiovascular diseases are controversial, and may depend on cellular source of S1P, S1P receptor expression and animal model used [38]. The relevance of endogenous S1P to atherosclerosis has not yet been investigated. Here, we confirm the critical role for hematopoietic S1P lyase, a key enzyme in intracellular S1P degradation and maintenance of S1P gradients in lymphoid organs, for lymphocyte trafficking. Moreover, we are the first to establish that hematopoietic S1P lyase deficiency leads to moderate monocytosis, possibly a resultant of G/GM-CSF dependent expansion of the myeloid lineage, to impaired chemokine-induced monocyte trafficking and activation, and to skewing of macrophage differentiation towards a more pro-inflammatory phenotype *in vitro* and *in vivo*. Collectively, these profound effects result in a reduced atherogenic response seen in LDL receptor knockout mice with hematopoietic *Sgpl1* deficiency.

Two mutually non-exclusive mechanisms may account for the diminished plaque development: a decreased lymphocyte availability and migratory activity on the one hand, and altered monocyte/macrophage migration and function on the other hand. The profound lymphopenia was a likely consequence of disruption of S1P gradients, which are crucial to normal lymphocyte egress from lymphoid organs. In this regard our observations largely recapitulate previous findings in S1P_1_ deficient mice [27] and after pharmacological S1P lyase inhibition by THI [11]. In the latter study, desensitization and subsequent downregulation of S1P_1_ was suggested to underlie the impaired T cell egress on exposure to high S1P concentrations. Our study failed to demonstrate a reduction in thymocyte S1P_1_ expression suggesting that S1P_1_ desensitization independent mechanisms may apply. Furthermore, reduced T cell mitogenic and migratory responses and decreased secretion of several T cell specific cytokines were reported *in vitro* in S1P-exposed lymphocytes and in LDLr^−/−^ mice treated with FTY720 [1]. The present study extends these findings to show impaired proliferation in *Sgpl1^−/−^* spleen lymphocytes and impaired homing capacity of spleen lymphocytes to spleen and lymph nodes, while *Sgpl1* deficient chimeras had aberrant spleen architecture, almost devoid of germinal centers. Additionally, the cytokine profiles of S1P lyase-deficient animals suggested attenuated T helper responses, as evidenced by decreased IL-2 (Th1) release upon CCL19 stimulation and by a CD4^+^/CD8^+^ dysbalance. Finally, the atheroprotective activity of hematopoietic S1P lyase deficiency might in part be ascribed to the expansion of CD4^+^CD25^+^ regulatory T (Treg) cells, known to exert potent anti-atherogenic effects [39], [40].

A second feature of S1P lyase-deficient chimeras was the mild monocytosis. In view of the increased responsiveness of *Sgpl1^−/−^* bone marrow cells to G/GM-CSF, this is likely caused by increased stromal production. In spite of elevated circulating monocyte numbers, plaque macrophage content remained unchanged in hematopoietic S1P lyase deficiency possibly due to an attenuated CCR2/MCP-1 chemotaxis. MCP-1 is not only required for stromal release but also for monocyte migration to inflammatory sites such as the plaque [32], [41]. MCP-1 levels in plasma and macrophage secretions of *Sgpl1^−/−^* chimeras were considerably reduced, while these chimeras also showed diminished peritoneal influx of CCR2-positive F4/80 macrophages in a model of MCP-1-dependent peritonitis, which points to aberrant macrophage migratory response. In this context, it is worth noting that reduced plasma MCP-1 levels were also observed in ApoE^−/−^ mice treated with FTY720 [22].

A few limitations of these studies should be mentioned: first, we applied a bone marrow setup to establish the effects of hematopoietic *Sgpl1^−/−^*deficiency instead of using a total body knockout mouse. However, *Sgpl1^−/−^* mice do not survive after 4 weeks, rendering long-term atherosclerosis studies impossible. In this study, we demonstrate that hematopoietic *Sgpl1* tightly regulates S1P gradients, illustrating the validity of the model used. Second, plaque T cell numbers were reduced, however when corrected for lesion size, the relative amount of plaque T cells is similar between the groups. It thus remains to be established whether the plaques are reduced caused by the reduction in T cell numbers or whether this is a secondary effect. The massive effects on T cell migration and proliferation suggest the former, but this has to be confirmed in the future. Furthermore, the exact mechanism and the contribution of each specific cell type, e.g. the role of regulatory T cells, to the observed reduction in atherosclerosis remains to be assessed.

In summary, hematopoietic S1P lyase appears to be essential for maintenance of S1P gradients and its absence not only has profound impact on lymphoid surveillance and lymphocyte activity, but also on stromal monocyte release and macrophage differentiation. Collectively, these pro- and anti-atherogenic effects of hematopoietic S1P lyase deficiency culminate in a dampened atherogenic response. Its broad activity profile on various key processes in adaptive and innate immunity suggests that any efforts to intervene with S1P lyase functioning as part of an anti-inflammatory or anti-atherosclerotic therapy should be considered with caution.
